# First-Year University Students’ Mental Health Trajectories Were Disrupted at the Onset of COVID-19, but Disruptions Were Not Linked to Housing and Financial Vulnerabilities: A Registered Report

**DOI:** 10.1177/21676968211053523

**Published:** 2021-11-01

**Authors:** Andrea L. Howard, Kendra D. Carnrite, Erin T. Barker

**Affiliations:** 1 Department of Psychology, 6339 Carleton University, Ottawa, ON, Canada

**Keywords:** transition to university, depression, anxiety, longitudinal, trajectories, COVID-19, food insecurity, low income, stress, living in residence

## Abstract

This study modeled disruptions in first-year undergraduates’ trajectories of mental health associated with the COVID-19 pandemic, testing whether disruptions were worse for students who moved residences, reported low family income, or were food insecure. Participants (*n *= 510) at a large Canadian university reported depression, anxiety, and stress in September, November, January, and March. In March 2020, in tandem with COVID-related campus closures, students also reported for each mental health measure whether their responses were influenced by personal experiences surrounding the pandemic. As hypothesized, students who reported feeling more COVID-related disruption reported poorer mental health in March. Contrary to hypotheses, mental health disruptions were not more pronounced for students who moved, had low income, or were food insecure. Survey administration at an early stage of COVID-19 combined with supports afforded by moving in with parents and near-universal government income assistance may have mitigated the incremental distress we hypothesized for vulnerable students.

The transition to university is a time of novelty, opportunity, and challenge for increasingly many emerging adults after leaving high school. Half of Canadian youth aged 17–21 were full- or part-time students in a postsecondary setting as of October 2019 (Statistics Canada, 2019)*,* and the U.S. Department of Education reports that 69% of high school graduates go on to postsecondary education (2020). In their first year, students face academic expectations and choices that differ vastly compared to high school; they encounter new social contexts, making new friends and leaving others behind; and many students move away from their parents’ home for the first time. Balancing and navigating this variety of new experiences can be initially overwhelming (Friedlander et al., 2007), and even high-achieving students can find themselves struggling to find their footing during this challenging transition (Schulenberg et al., 2004). The transition to adulthood in general is a peak period for onset of mental disorders (Pearson et al., 2013; Rohde et al., 2013) and in a typical year, close to one third of first-year undergraduates meet screening criteria for a depressive, anxiety, or substance use disorder (Auerbach et al., 2018).

Unlike any typical academic year in recent history, students who matriculated in 2019 faced an abrupt interruption to their first year of university in March 2020, when the novel coronavirus disease (COVID-19) rapidly shut down campuses and effectively cancelled day-to-day student life. Classes pivoted immediately online; campuses emptied and many students living in residence were asked to leave; libraries and other campus resources—including mental health supports—were disrupted or became unavailable. Social isolation quickly became a new public mandate as many cities locked down, preventing students from physically interacting with people outside of their households. Changes of this magnitude raise immediate concerns for student mental health given extensive evidence that negative life events and stressors worsen symptoms of depression and anxiety (e.g., Breslau et al., 1991; Hazel et al., 2008; Schilling et al., 2007; Turner & Butler, 2003). Early evidence from the COVID-19 pandemic suggests that students’ mental health was dramatically impacted. For example, depression, anxiety, and suicidal thoughts all increased among Greek undergraduates during lockdown (Kaparounaki et al., 2020), as did stress and anxiety levels in studies assessing the mental health of university students in lockdown in France (Husky et al., 2020) and the United States (Browning et al., 2021).

Given the rapid transformation of student life brought on by COVID-19, the aim of the present study was to estimate the level of disruption in first-year students’ typical trajectories of depression, anxiety, and stress attributable to the circumstances of the COVID-19 pandemic. We drew on data from a four-wave longitudinal study of Canadian undergraduates that began in September 2019 and completed its final follow-up in late March 2020—at the peak of first-wave COVID-19 escalation and shortly after campuses were closed to in-person instruction.

## Student Mental Health

Mental health difficulties are common among college and university students, with around one in four Canadian undergraduates self-reporting a past-year diagnosis of depression, one in five reporting past-year anxiety, and 46% reporting heightened stress levels (ACHA, 2019). Internationally, the pooled 12-month prevalence of any mood, anxiety, or substance use disorder was 31.4% based on diagnostic screening surveys of first-year undergraduates in 8 countries (Auerbach et al., 2018). Other data suggest that this prevalence pattern is not new. Among college students in the nationally representative 2001–2002 U.S. NESARC sample, 40% presented with a mood, anxiety, or substance use disorder in the past 12 months (Blanco et al., 2008). Data collected from counseling centers on U.S. campuses from 2010 to 2015 suggest that modest upward trends in depression, anxiety, and distress as well as increased treatment-seeking for suicidal ideation in part reflect years of sustained efforts to reduce stigma and encourage help-seeking (Xiao et al., 2017). However, campuses worldwide have struggled to keep up with demand for mental health services. Each year, the numbers of students presenting with psychological disorders that require care increase, often without commensurate increases in staffing and other resources to meet demand (LeViness et al., 2019). At the rates observed in recent years, poor mental health is a significant threat to students’ academic success and long-term well-being.

Poor mental health in students has been linked to sleep problems, poor exercise habits, substance use, and poorer social and academic functioning (Alonso et al., 2018; Bruffaerts et al., 2018; Doom & Haeffel, 2013; Stallman, 2010). Students experiencing poorer mental health also tend to earn lower grades (Bruffaerts et al., 2018) and are less likely to complete their degrees (Kessler et al., 1995). One study showed that students with elevated depressive symptoms during their first semester were more likely to drop out by the end of their second year (Boyraz et al., 2016). Considering the employment and income advantages associated with completing an undergraduate degree (Frenette, 2017; U.S. DoE, 2020), students attending postsecondary while experiencing poor mental health are at greater risk of long-term employment deficits. This risk begins early, as signs of worsening mental health are immediately evident in students making the transition to university. Studies that assessed students in the months or weeks leading up to the start of their first year show substantial increases in symptoms of depression and anxiety within the first weeks of the Fall semester (Cooke et al., 2006; Doane et al., 2015; Levine et al., 2020). In the academic year ending in 2020, COVID-19 may have amplified mental health risk for a larger proportion of students than usual.

## Tracking Mental Health Across the Transition to University

Enrolling in college or university bears many characteristics of a transition-linked turning point (Graber & Brooks-Gunn, 1996) capable of producing developmental change that may have lifelong implications. In particular, we expect that many first-year students find the simultaneous changes in multiple domains of functioning to be taxing, likely accounting for some of the sudden intensification of mental health symptoms after arriving on campus. At the same time, emerging adults in and out of postsecondary education exhibit high rates of diagnosable mental illness, and students tend to fare similarly or better than their non-student age mates (Auerbach et al., 2016; Blanco et al., 2008; Wiens et al., 2020). Longitudinal assessments across the transition to university are needed to clarify how extensively the challenges of the first year persist.

In several studies, symptoms of ill mental health remained stable on average across students’ first semester (Finkelstein-Fox et al., 2018) and through the Spring of students’ first year (Azmitia et al., 2013; Hirai et al., 2015; Lee et al., 2014; Levine et al., 2020). Others show that depression worsens from the beginning to the end of the first semester (Howard et al., 2020) before improving toward the end of the first year (Barker et al., 2018; Cooke et al., 2006). In the longer term, well-being tends to improve on average as emerging adults make their way through their twenties (Galambos et al., 2006; Galambos & Krahn, 2008; Gao et al., 2020). Within the context of the first year, however, available evidence suggests that on average, students do not return to their pre-college symptom levels. At the same time, average trends disguise considerable between-person variability comprising a range of trajectories from flourishing to floundering. In the context of the COVID-19 pandemic, the outcomes of students with additional vulnerabilities are especially concerning. In the present study, we focus on students whose living situations were disrupted during campus closures, and students who are more financially vulnerable (students reporting lower family incomes and students reporting food insecurity).

## Living Situation Vulnerability

Many emerging adults begin to live independently or semi-independently for the first time during their first year of university, and the decision to live on- or off-campus is linked to long-term health and academic functioning. Students living off-campus tend to maintain better physical health than their on-campus counterparts (Henry et al., 2018). However, living in a residence hall carries more academic and mental health advantages for students. Students living on-campus drop out less often (Bozick, 2007) and have fewer symptoms of anxiety and depression (Eisenberg et al., 2007, 2013; Stallman, 2010). These benefits may stem from limited domestic responsibilities (e.g., cooking and cleaning) and the readily available campus resources (e.g., meal plans and mental health services) found in residences (Beiter et al., 2015). Living on-campus may also facilitate more friendships (Buote et al., 2007), engendering feelings of belongingness to campus (Maestas et al., 2007). Studies show that greater campus belongingness is related to academic persistence (Gopalan & Brady, 2020) and competence (Pittman & Richmond, 2008), suggesting more academic advantages for students living in residence halls. However, as students typically do not live on-campus long-term (Amole, 2009), the benefits associated with residence life may diminish as students transition off-campus.

Longitudinal studies examining changes in student living situations link residential mobility to lower academic retention rates (Swanson & Schneider, 1999), and lower quality social relationships (Vernberg, 1990). Students experiencing more disruptions in their living situations also report poorer mental health (Copp et al., 2017). Specifically, students who *transition from independent to dependent living situations* report more depressive symptoms relative to students whose living arrangements remain stable (e.g., living consistently independently or consistently dependently; Copp et al., 2017). This suggests that students moving back into a parental home (and thus back to more dependent living) may be more vulnerable to worsening mental health. The COVID-19 pandemic brought about a mass disruption to student living situations, with many students leaving campus residences to move home with family. In the present study, we test whether students whose living situations abruptly changed near the end of the academic year experienced worse mental health disruption compared to their peers whose living situations remained stable.

## Financial Vulnerability

Income disparities are a persistent challenge in access to postsecondary education. Half of Canadian undergraduates and two thirds of U.S. undergraduates finish their degrees with student debt, averaging $28,000 (CUSC-CCREU, 2018) and $31,800 (U.S. DoE, 2020), respectively. Students experiencing financial strain are less likely to complete their degrees than their peers from higher-income families and peers carrying less debt (Joo et al., 2009). Unsurprisingly, students coming from lower-income families report more financial stress and student debt than students from higher-income backgrounds (Houle, 2014), and financial stress is associated with more symptoms of depression, anxiety, and stress (Richardson et al., 2017). In a large sample of students in all years of study, 22% showed profiles of risk characterized by financial stress, low social support, and histories of stress that included low SES (Newcomb-Anjo et al., 2017). These students had the highest levels of depressive symptoms, anxiety, and academic stress compared to other groups.

Students experiencing financial strain face difficult budgeting decisions to ensure their tuition and fees are paid. In some cases, this means reducing or foregoing spending on basic needs. Indeed, up to 40% of Canadian undergraduates (e.g., Entz et al., 2017; Olauson et al., 2017; Blundell et al., 2019) and over half of U.S. undergraduates (e.g., Broton & Goldrick-Rab, 2017) report at least some *food insecurity* (includes anxiety or worries about running out of food, making compromises in food quality because of limited funds, and skipping meals or going full days without food). Higher rates of food insecurity are prevalent in students who are financially independent and receive no financial resources from family members (Bruening et al., 2016).

The burdens of limited income and food insecurity present substantial concerns for students’ academic success and mental health. Economically disadvantaged and food insecure students have more academic difficulties, are at higher risk of dropping out (Bozick, 2007; Payne-Sturges et al., 2018), and report more depressive symptoms and stress (Eisenberg et al., 2013; Payne-Sturges et al., 2018; Stebleton et al., 2014). Financial vulnerabilities take their toll even in students’ first year of university. In the sample we drew on for the present study, 15.7% reported moderate or severe food insecurity in September along with more symptoms of depression, anxiety, and stress (Howard & Barker, 2021) compared to their food secure peers. Given that these assessments were completed within the first three weeks of classes, we can infer that financially strained students were already vulnerable to poorer mental health before the COVID-19 pandemic. The resulting economic depression and student job losses lead us to suspect that the impacts of COVID-19 on students’ first-year mental health trajectory may be intensified for financially vulnerable students, a question we tested in the present study with separate measures of family income and food insecurity.

### The Current Study

We drew on data from a four-wave longitudinal study of first-year undergraduates at a large Canadian university that began in September 2019 and completed its final follow-up in late March 2020^1^. The first aim was to estimate the level of disruption in first-year students’ typical trajectories of depression, anxiety, and stress that couldcoulcould be attributed to perceptions surrounding the COVID-19 pandemic. We accomplished this by modeling year-long trajectories of each measure and estimating the time-specific effect of responses in March to a question asking how much students felt that their responses to the measure were “influenced by feelings, experiences, and reactions you are having” to the COVID-19 pandemic. We posed the following hypotheses:

Hypothesis 1a Mean trajectories of depression, anxiety, and stress will increase from September to the end of the first semester and remain elevated into the new year.

Hypothesis 1b Mental health of students reporting stronger COVID-related influences on their responses will maintain or worsen at the end of the year. In contrast, trajectories for students reporting weaker COVID-related influences will follow patterns typically seen in other research and show improvements in mental health at the end of the year.

The second aim of this study was to test whether COVID-related disruptions to first-year students’ trajectories of depression, anxiety, and stress are worse for students for whom living arrangements were disrupted and for students who began their academic year in a position of financial vulnerability (food insecure; lower family income). We accomplished this by testing interactions between each vulnerability and students’ COVID-19 response. We posed the following hypotheses:

Hypothesis 2a Mean trajectories will be similar for students living with parents, in residence, and off-campus prior to COVID-19.

Hypothesis 2b End-of-semester disruptions to mental health will be worse for students who moved residences in March—most of whom should be moving out of campus residence and back into parents’ homes—compared to students who did not move residences.

Hypothesis 2c Mental health disruptions will be worse for students from lower-income families and for students reporting food insecurity.

Finally, as an exploratory question, we tested whether the effects of living situation and financial vulnerability were additive or multiplicative by including interactions between each of these measures in models predicting COVID-related disruptions to end-of-semester depression, anxiety, and stress (Exploratory Aim 1).

## Method

### Participants and Procedure

This study drew on existing data collected from first-year undergraduate students at a large Canadian university who completed online surveys aimed at understanding success and well-being during the transition to university. Participants were recruited during orientation week for first-year students in September 2019. Undergraduate recruiters handed out postcards and candy across campus, inviting students to complete online eligibility screening questions. Students were eligible if they declared that this was their first year studying at any college or university (excepting CEGEP, or Grade 12/13 “junior college” for students from Québec), that they were studying full-time, and that they were under 20 years old. Ineligible students were thanked for their time and directed out of the screening survey. Eligible students were invited to provide their name and email address and were contacted with an invitation and a personalized link to the intake survey. We received 1102 responses to an initial eligibility screening survey, of which *n *= 600 were eligible to participate and chose to provide a name and email address. Of those, *n *= 517 initiated an intake survey. Five people were excluded because they subsequently failed to meet age eligibility criteria and two were excluded due to an incomplete demographics section with no other questions answered. The final sample size was *n*=510, for a participation rate of 85% of eligible screened respondents with unique contact information.

Students who completed the intake survey received a $10 *amazon.ca* gift code and were entered into a draw to win a $100 *amazon.ca* gift code. Most participants (*n *= 481) consented to participate in our year-long longitudinal study and these students were re-contacted in November 2019 and in January and March 2020 to complete follow-up surveys. Participation rates were *n *= 408 (84.8%), *n *= 382 (79.4%), and *n *= 411 (85.4%) in November, January, and March, respectively. We offered a $10 gift code per survey and a $10 bonus to students who completed all four surveys. To boost retention in March we increased the compensation for that survey to $20.

### Measures

#### Sociodemographic characteristics at intake

Students provided their age, whether they were a first-generation student, whether they were an international student, whether they grew up in a two-parent family, and their parents’ combined past-year income. Students also provided their gender identity but this was not collected at intake due to a survey error and is incomplete for 68 participants (13.3%).

#### Mental health

The Center for Epidemiologic Studies Depression Scale (CES-D; Radloff, 1977) assessed *depressive symptoms *at each wave. Participants responded to 10 statements based on how often they felt similar to the prompt during the past 2 weeks (e.g., “I was bothered by things that don’t usually bother me”), on a 4-point scale: 0 (rarely or none of the time [less than 2 days]), 1 (some or a little of the time [2–5 days]), 2 (occasionally or a moderate amount of time [6–9 days]), and 3 (most or all of the time [10–14 days]). The CES-D-10 scale has been validated in late adolescents (Bradley et al., 2010), clinical (Björgvinsson et al., 2013), and older populations (Andresen et al., 1994), and has been found to be comparable to the well-validated, full-length CES-D scale (Andresen et al., 1994).

The GAD-7 (Spitzer et al., 2006) assessed *anxiety symptoms* at each wave. Participants indicated how often they were bothered by each of seven problems during the past 2 weeks (e.g., “worrying too much about different things”; “feeling nervous, anxious, or on edge”), rated on the same scale as the CES-D. The GAD-7 scale has been shown to be valid in both clinical (Spitzer et al., 2006) and general population samples (Löwe et al., 2008).

We administered the 4-item version of the Perceived Stress Scale (Cohen et al., 1983) to measure *stress* at each assessment. Participants were asked about thoughts and feelings during the past 2 weeks and indicated how often each statement applied to them (e.g., “how often have you felt that difficulties were piling up so high that you could not overcome them?”; “how often have you felt confident about your ability to handle your personal problems?” [reverse coded]). Ratings were given on a 5-point scale with anchor points 1 (*never*), 2 (*almost never*), 3 (*sometimes*), 4 (fairly often), and 5 (*very often*). The PSS-4 scale has shown adequate psychometric properties in the general population (Cohen & Williamson, 1988), and in multiple countries (Lesage et al., 2012; Leung et al., 2010).

In our prior research with these measures, each scale has tended to exhibit unidimensionality and consistent, high factor loadings. Thus, our preregistered plan was to compute a mean score for each measure at each assessment based on a factor structure verified prior to scale scoring. Item sets were considered adequate for mean scoring if they produced results in exploratory factor analyses favoring one-factor solutions and produced standardized factor loadings in confirmatory factor analyses that exceeded .60. Our plan for instruments whose properties were not as expected (e.g., multidimensionality, locally dependent item sets, and unusually low factor loadings) was to explore ad-hoc modifications such as using a subset of items that do support unidimensionality or computing factor scores instead of mean scores if item loadings were highly variable.

#### Early impact of COVID-19

In the two weeks prior to the planned release of the March follow-up assessment, it became clear that the SARS Cov2 virus had made its way to Canada and was circulating largely unimpeded in the community. Our campus community was informed on Friday, March 13, 2020 that all instruction would be delivered remotely beginning March 18. With the approval of our Institutional Review Board, our team quickly added new questions to our survey in an effort to capture—and adjust for—any systematic effects of COVID-19 on students’ responses.

We were primarily concerned that our mental health measures might be compromised. At the bottom of each relevant questionnaire (depression, anxiety, and stress), we added the following item: “How much do you feel that your responses to the questions above are influenced by feelings, experiences, and reactions you are having to the Coronavirus (COVID-19) pandemic?” Response options were on a 4-point scale with anchor points 1 (*not at all*), 2 (*somewhat*), 3 (*moderately*), and 4 (*very much*). Thus, early impact of COVID-19 was measured separately for each mental health instrument.

#### Living arrangements

At each assessment, students indicated whether they were living with parents, living on-campus, or living off-campus. The final assessment was distributed on March 21st, one week after university administration cancelled in-person classes and encouraged—but did not require—students to move out if possible. Students living in residence were given a deadline of March 22nd to move out if they wished to receive a pro-rated refund on their room and board costs for the remainder of the semester. From these data, we constructed two measures of living arrangements: (1) pre-COVID-19 contrast-coded variables differentiating students living with parents (coded 0.67) versus away from parents (coded −0.33); and students living in campus residence (coded 0.5) versus in other off-campus accommodations (coded −0.5) based on their most recent report prior to the March follow-up survey, and (2) a dummy-coded variable identifying students who changed accommodations (e.g., moved out of residence and back to parents’ home, coded 1) versus did not change since their most recent prior report (coded 0).

#### Financial vulnerability

Parents’ combined past-year income was measured using a single item with response options ranging from 1 (less than $5000) to 12 ($200,000 or greater). A second index of financial vulnerability was food insecurity. Students completed the 10-item *Canadian Household Food Security Survey Module* (Health Canada, 2012) at the September intake. This module is a screening tool, so participants only see additional questions if they respond affirmatively to at least one of three initial questions (worries about running out of food; ran out of money to replenish food; and unable to afford to eat balanced meals). Subsequent questions inquire about cutting portion sizes or skipping meals, going hungry, losing weight, and not eating for a whole day because there wasn’t enough money for food. Students in this study were classified as *food insecure* if they met criteria for at least moderate food insecurity (responding affirmatively to at least 2 questions). We previously used this variable (Howard & Barker, 2021) and classified 15.7% of students (*n*=77/491) as food insecure.

### Analysis Strategy

Analysis plans for this study were preregistered in a Stage 1 report available at https://osf.io/2dpkx. We used multilevel linear modeling to estimate rates of change over an eight-month academic year in reported symptoms of depression, anxiety, and stress (Hypothesis 1a). Four assessment waves allowed us to consider linear and curvilinear trends in growth over the year, and the optimal form of change was selected prior to including other measures in our models. Our preregistered plan to select an optimal functional form was to compare quadratic and linear change models, and to visually inspect students’ raw and predicted trajectories of change. Criteria for selecting a quadratic change model were twofold: (1) if a likelihood ratio test supported the model with quadratic growth, and (2) if the difference between the vertex (peak of the mean quadratic trajectory) and the start or end of the trajectory—whichever is larger—was greater than the span of the 95% confidence interval around the slope estimate from the linear change model. These criteria were selected to guard against overfitting the data when curvilinearity is statistically significant but not visually prominent in plotted data. Null hypothesis significance tests of the linear and/or quadratic change parameters were used to infer whether mean change increased, decreased, or remained stable.

Trajectories estimated using this procedure reflected the course of mental health across an academic year that included COVID-19 disruption at its final time point. We estimated the contribution of this disruption by including a time-varying covariate comprising students’ responses to the relevant “early impact of COVID-19” question at the final assessment point and zeroes at all other assessments. Null hypothesis significance tests of this effect for each of depression, anxiety, and stress were used to infer whether COVID-19 disruption was associated with poorer mental health at the end of students’ first year (Hypothesis 1b).

Model testing proceeded sequentially. Coded variables for student changes in living situation were added next as person-level predictors of levels and rates of change in mental health across the academic year. Null hypothesis significance tests of these predictors for each of depression, anxiety, and stress were used to infer whether mean trajectories were similar or different for students living with parents, in residence, and off-campus prior to COVID-19 (Hypothesis 2a). Adding an interaction between living situation and the COVID-19 impact covariate tested whether disruptions to mental health were worse for students who moved residences in March (Hypothesis 2b). Adding measures of food insecurity and parents’ combined income and their interaction with the COVID-19 impact covariate tested whether disruptions to mental health were worse for students from lower-income families and for students reporting food insecurity (Hypothesis 2c). Finally, we added three-way interactions of living situation × financial vulnerability × COVID-19 impact to test Exploratory Aim 1. Adjustments for multiple comparisons across many inferential tests are noted below.

Model steps testing each hypothesis also included relevant sociodemographic covariates as correlates of level, but not change, in mental health measures, namely: age, first-generation student status, international student status, and whether students grew up in a two-parent family (vs. single parent). Models at all sequential steps listed above were tested with and without covariates. The model without covariates is reported in full in our supplemental materials, as are sample analysis commands with an artificial dataset that we prepared for our Stage 1 report (https://osf.io/sbmtg).

#### Adjustment for multiple comparisons, model sensitivity, and results reporting

We adjusted for Type I error inflation separately in each model for mental health (depression, anxiety, and stress) using the Benjamini–Hochberg False Discovery Rate procedure (Benjamini & Hochberg, 1995) against a nominal alpha of .05. Any interactions found to be statistically significant were tested again in models that excluded other interactions to check the stability of the findings. Unstandardized effects, standard errors, confidence intervals, and *p*-values are reported in tables. Our preregistered plan was to plot fitted trajectories for average trends, effects of COVID-19 impact, and statistically significant interactions with confidence regions, and to probe interactions to determine regions of significance for residential and financial vulnerability moderation (Bauer & Curran, 2005; Preacher et al., 2006).

#### Power and effect size

The budget for this longitudinal data collection was set based on power requirements for a separate research focus requiring more complexity than the analyses proposed in the present study. Consequently, the sample is overpowered to detect simple longitudinal trends and trivially small changes over time were likely to be flagged as statistically significant. We probed the scope of this problem in two simulation studies (see Supplement 2, https://osf.io/sbmtg).

In the first study, we simulated longitudinal data exhibiting no change over time to establish confidence limits around a trajectory of zero change over time and identify slope values that are consistent with zero change. We used R software (R Core Team, 2019) to simulate a dataset comprising *n *= 510 cases at an initial intake assessment and introduced randomly missing values at three subsequent assessments in proportions matching the rate of missing in our data (missForest package; Stekhoven & Bühlmann, 2012; Stekhoven, 2013). The repeated measure of interest was simulated from a normal distribution with sample statistics consistent with measures of depression and anxiety we used in the present study and have used in the past (*M *= 1 and *SD*s ranging from .5 to .8 on a 0 to 3 scale). In this simulation study, scores at each wave were uncorrelated, meaning that on average, a test of change over time for a mixed model should return a fixed effect of *time* equal to zero (we used the lme4 package; Bates et al., 2015). We repeated this data simulation process 1000 times and saved fixed effects of time from each model. As expected, around 5% of data sets produced time effects that were significantly different from zero. Simulations with smaller *SD*s on our repeated measures produced narrower confidence limits on the fixed effects of time than simulations with larger *SD*s in our repeated measures. Even in the latter scenario (*SD *= .8), the 95% confidence interval of a zero-change trajectory was −.035 to .030 (see Supplement 2, “Simulation 1”, for syntax and results).

In the second study, we repeated the process described above but simulated a nonzero slope. We found that a very small *time* effect—corresponding to linear change of just .10 units per wave on a 0 to 3 scale—was statistically significant >99% of the time (see “Simulation 2” in Supplement 2). Given these findings, it was likely that even a small divergence from the mean growth trajectory at the end of the year would be statistically significant, permitting us to easily conclude that COVID-19 changes were associated with mental health disruption. Consequently, we chose to define a benchmark *effect size of interest *for the present study. To be meaningful, we reasoned that the perceived impact of COVID-19 on students’ mental health at the end of the semester ought to be at least as large as the size of worsening mental health associated with entering university. We consulted studies cited earlier that assessed students in the months or weeks leading up to the start of their first year (Cooke et al., 2006; Doane et al., 2015; Levine et al., 2020). Using summary statistics of mental health measurements taken before and after the start of the first year in these studies (depression, anxiety, and stress), we calculated an average effect equal to a standardized mean difference of 0.29. On a scale ranging from 0 to 3 with a *SD* of .8, for example, this translates to a raw difference of 0.23 units—about one quarter of the distance between two points on the Likert scale. For the purposes of the present study, this served as the target effect size of interest used to evaluate the magnitude of COVID-related disruption in mental health at the end of the year, and we used equivalence testing (e.g., Lakens et al., 2018) to determine whether the confidence interval for the effect of COVID-19 disruption met, exceeded, or fell below this benchmark.

Following the same procedures as above, we simulated a dataset exhibiting modest curvilinear change in symptoms across four time points and introduced a “COVID-19 effect” at the final time point equal to the benchmark effect size (see “Simulation 3” in Supplement 2). Across 1000 replications, this effect was statistically significant (*p*<.05) in all samples (empirical power >99%), with a mean raw effect of .30 and 95% confidence interval of .22–.39. Our design was thus highly-powered for this planned analysis.

#### Missing data handling

Our preregistered plan was to compare cases with complete versus incomplete longitudinal data on relevant sociodemographic and mental health measures collected at the September assessment. This approach allowed us to identify any variables that explain missingness and include them as covariates in our analysis to improve the plausibility of the missing at random (MAR) assumption required for full information maximum likelihood (FIML) data analysis.

## Results

Unless explicitly stated, all analyses reported below were preregistered. Means, *SD*s, percentages, and sample sizes are presented in Tables 1 and 2. A complete table of correlations among all measures used in this study is available in the Supplemental files (https://osf.io/sbmtg). The sample was gender balanced, with ages ranging from 16.4 to 19.9. Participants were ethnically diverse: 50.1% self-identified as White, 13.9% as South Asian, 10.4% as Southeast Asian, 6.1% as Black, 5.7% as West Asian/Middle Eastern, 2.7% as Indigenous (First Nations, Métis, or Inuit), 2.4% as Latinx, 7.6% as multiple ethnicities, and 0.6% as another ethnicity.

**Table 1.** Means, Standard Deviations (SD), and Valid Responses (*n*) for Continuous Variables.

	Mean	SD	*n*
Depression			
September	1.03	0.61	484
Early December	1.27	0.67	388
Late January	1.26	0.66	366
March	1.35	0.67	396
Anxiety			
September	1.07	0.79	484
Early December	1.33	0.84	386
Late January	1.29	0.82	365
March	1.32	0.85	396
Stress			
September	1.83	0.73	483
Early December	2.04	0.79	387
Late January	1.95	0.73	365
March	2.20	0.77	396
Early Impact COVID-19			
Depression	2.62	0.99	390
Anxiety	2.64	1.03	395
Stress	2.54	1.04	396
Parent income	8.43	2.62	472
Age in years	18.34	0.50	504

**Table 2.** Percent (%) and Valid Responses (*n*) for Categorical Variables.

	%	*n*
Living arrangements		
With parents	35.3	180
In residence	54.7	279
Off-campus	10.0	51
Moved in March	40.2	205
Food insecure	15.7	77
International student	8.2	42
First generation	22.0	112
Two-parent family	82.8	419
Gender identity		
Female	54.7	242
Male	44.6	197
Neither	0.7	3

Predictor variables and covariates were inspected for heteroscedasticity and multilevel diagnostic measures were used to identify any potential influential cases. Several cases with high Cook’s distance values or at least two extreme *DFBETAS* associated with coefficients in the final models were flagged. Results were unaffected by the presence of flagged cases in the data, and findings reported below draw on the complete data.

### Mental Health Factor Structure

For depression and anxiety, exploratory factor analyses confirmed that single-factor structures were well-supported for item sets gathered at each wave of assessment, and our longitudinal analyses used mean scores as planned. For stress, factor analyses suggested only adequate support for a one-factor structure with the two positively-worded items achieving weaker loadings than the two negatively-worded items. We suspected that this reflected local dependence between the same-valenced item pairs. A one-factor confirmatory analysis verified this suspicion and a residual correlation between the two positively-worded items eliminated all model mis-fit. Our final stress measure used extracted factor scores from a longitudinal confirmatory factor analysis in which the positively-worded item pairs were permitted a residual correlation within each wave and each of the four latent factors capturing stress were freely correlated with the other factors.

This model provided strong fit to the stress data (χ^2^ (70) = 143.36, RMSEA = .046, *CI*_*95%*_ [.035, .057], CFI = .966, SRMR = .059). Note that our preregistered plan allowed for the possibility of computing factor scores if necessary, but this specific modification was devised only after examining the data.

### Missing Data

A detailed inspection of available data showed that *n *= 487 students (95% of the original sample) responded to our depression, anxiety, and stress instruments at least once, and *n *= 331 (65% of the original sample) completed all four waves. The remaining participants completed three waves (*n *= 56) or two waves (*n *= 42). Cases with complete versus incomplete data did not differ on any baseline demographic or mental health measures, increasing our confidence in invoking the MAR assumption for the present analyses.

We made two key adjustments to our preregistered analysis plan to minimize listwise deletion associated with missing values present in predictor variables and covariates. First, gender identity was missing for 13.3% of participants (*n *= 68) due to a survey error. Our analysis models included two coded variables to capture Male (1) versus Female (0) and Neither/unknown (1) versus Female (0). Based on records of participants’ names, we imputed values for Male or Female if a participant’s first name was associated with a single gender in at least 95% of the records for baby names registered in the Province of Ontario between 1999 and 2003 (birth years consistent with the ages of participants in the present study; Government of Ontario, 2020a; 2020b). For example, of the 1007 children named “Melissa”, 100% were assigned a female gender, and none were assigned a male gender at birth. A participant reporting this name would be assigned a code of 0 for Female for the purposes of analysis. In contrast, of the 171 children named “Robin”, 58% were assigned a female gender and 42% a male gender at birth. A participant reporting this name would be assigned a code of 1 for Neither/unknown for the purposes of analysis. We were able to assign Male/Female gender codes to 54 participants this way, and 14 were assigned Neither/unknown. Crucially, we used this approach strictly to preserve data in the analysis and do not presume that the codes assigned necessarily match all missing participants’ gender identities. Descriptive statistics reported in Table 2 show only gender identities directly disclosed in our study (no participants declined to report a gender identity).

Second, we noted that 97 participants were missing in March, the wave at which participants completed their early impact of COVID-19 ratings. For these participants, we assigned a COVID-19 impact score of 0 and included a variable representing cases who were present (coded 0) versus missing (coded 1) in March to prevent listwise deletion (a simple *pattern mixture* approach; see Hedeker & Gibbons, 1997).

### Hypothesis 1a: Patterns of Change in Mental Health

For depression and anxiety symptoms, a quadratic function was selected as the best fitting form of change across the year. Likelihood ratio tests supported these models over simpler linear change (for depression: *χ*^2^ (1) = 14.51, *p* =.00014; for anxiety: *χ*^2^ (1) = 20.91, *p* <.0001). In both cases, the strong linear increase meant that the difference between the peak of the quadratic curve and the starting value of symptoms in September was many times larger than the width of the confidence interval around the slope estimate from the linear change model, satisfying our second criterion for including quadratic trends in the model. We reconsidered this strategy given that any trivially small curve would meet this criterion in the presence of a strong linear trend. Instead, we drew a straight line between the starting and ending points of the fitted quadratic curve and computed the difference between each point on the curve and its corresponding point on the straight line. For each of depression and anxiety symptom trajectories, the largest difference between curve and line was still over twice as large as the width of the 95% confidence interval around the slope estimate from the linear change model (see the Supplemental files for R code showing these calculations, https://osf.io/sbmtg). Quadratic change was thus firmly supported by our revised criterion, and we retained this form in all subsequent models for depression and anxiety. For stress, a likelihood ratio test did not show improvement for a model with quadratic change (*χ*^2^ (1) = .51, *p* =.474), and the linear change model was retained.

As hypothesized, the mean trajectories of depression and anxiety symptoms increased from September to the end of the first semester and remained elevated thereafter. The mean trajectory of stress increased across the academic year. Fixed effects from these models are given in Table 3. Final results for each outcome showing models after removal of non-significant interaction terms are given in Table 4. A complete summary of all model-building steps, including models tested with and without covariates, appear in the Supplemental files (https://osf.io/sbmtg).

**Table 3.** Fixed Effects of Unconditional Multilevel Linear Models Showing Change in Depression, Anxiety, and Stress from September 2019 to March 2020.

	Without Covariates		With Covariates	
	*Est (SE)*	*CI*_*95%*_ (LL,UL)	*Est (SE)*	*CI*_*95%*_ (LL,UL)
Depression				
Intercept	1.04 (.03)	(.98, 1.10)	1.17 (.04)	(1.09, 1.25)
Time	.21 (.03)	(.15, .28)	.20 (.03)	(.14, .26)
Time^2^	−.04 (.01)	(−.06, −.02)	−.03 (.01)	(−.05, −.02)
Anxiety				
Intercept	1.09 (.04)	(1.01,1.16)	1.28 (.05)	(1.18, 1.38)
Time	.24 (.04)	(.16,.31)	.22 (.04)	(.15, .30)
Time^2^	−.06 (.01)	(−.08,−.03)	−.05 (.01)	(−.07, −.03)
Stress				
Intercept	1.85 (.03)	(1.78, 1.91)	2.03 (.05)	(1.94, 2.12)
Time	.10 (.01)	(.08, .12)	.10 (.01)	(.08, .12)

*Note*. All effects are significantly different from zero (largest *p *= .0005). The model with covariates includes participant age, international student status, first-generation student status, whether participants grew up in single-parent families, male gender, and neither/unknown gender. Est = Fixed effect estimate. SE = Standard error. *CI*_*95%*_ = 95% Confidence Interval around the estimate. LL = lower confidence limit, UL = upper confidence limit.

**Table 4.** Results of Final Multilevel Linear Models for Change in Depression, Anxiety, and Stress.

	Depression	Anxiety	Stress
	*Est (LL, UL)*	*Est (LL, UL)*	*Est (LL, UL)*
Intercept	1.10 (.95,1.25)***	1.22 (1.03, 1.41)***	1.94 (1.77, 2.11)***
Time	.37 (.27, .47)***	.42 (.30, .54)***	.06 (.02, .09)**
Time^2^	−**.13 (**−**.18,**−.08)***	−**.16 (**−**.22,** −.11)***	-
COVID-19 Wave	.06 (−.13,.25)	.09 (−.15, .32)	−.14 (−.26, −.02)
Perceived COVID-19 impact	.02 (−.04, .07)	.05 (−.01, .12)	.06 (−.003, .12)
Wave x Impact	.20 (.15,.25)***	.23 (.17, .29)***	.19 (.13, .24)***
Living away from parents	−.03 (−.18, .12)	.03 (−.16, .22)	−.08 (−.26, .10)
Living in residence	−.03 (−.20, .14)	.02 (−.20, .24)	.09 (−.11, .29)
Moved in March	−.03 (−.18, .12)	−.14 (−.33, .05)	−.10 (−.27, .07)
Food insecure	.32 (.18, .46)***	.25 (.07, .43)**	.33 (.17, .50)***
Parent income	−.01 (−.03, .01)	−.01 (−.04, .01)	−.01 (−.03, .02)
Covariates			
Missing data in March	.07 (−.10, .24)	.08 (−.15, .30)	.03 (−.17, .24)
Age	.06 (−.03, .16)	.004 (−.12, .13)	.02 (−.10, .13)
Male gender	−**.27 (**−**.37,** −.17)***	−**.41 (**−**.53,** −.28)***	−**.35 (**−**.47,** −.24)***
Neither/unknown gender	−.34 (−.70, .01)	−.37 (−.83, .08)	−.26 (−.68, .15)
International student	−.13 (−.33, .07)	−.34 (-.60, -.09)**	−.18 (−.42, .05)
First generation student	−.02 (−.14, .09)	−.04 (−.19, .11)	−.05 (−.19, .09)
One-parent family	.08 (−.05, .22)	.09 (−.09, .26)	.03 (−.12, .19)

*Note*. Effects flagged as statistically significant after correction for multiple testing highlighted in **bold**. COVID-19 Wave = Time-varying covariate coded 1 in March and coded 0 in all other months. Age and parent income are centered at their respective sample means. Simple slopes describing effects of COVID-19 impact in March based on the Wave x Impact interaction and associated terms are reported in the article text.

***p < .001, **p < .01, *p < .05.

### Hypothesis 1b: Early Impact of COVID-19 on Mental Health Trajectories

For each outcome, three terms were needed to test the contribution of COVID-related influences on students’ ratings in March of their mental health. This deviates slightly from what we preregistered in the Stage 1 report but is more accurate than our original single-covariate plan. First, a dummy code differentiated the first three time points (coded 0) from the March time point (coded 1). Second, the “early impact of COVID-19” score was added as a person-level predictor. Third, a cross-level interaction was formed between the dummy code and the COVID-19 early impact score. This term, followed up with a simple slope of the COVID-19 effect in March, provides a test of Hypothesis 1b.

Hypothesis 1b was supported. There were significant and positive effects of COVID-19 scores on all mental health outcomes in March. For depression, the simple slope was .22 (*SE* = .03; *CI*_*95%*_ = .16, .29; *p*<.0001), with standardized effect size of .33 (*CI*_*95%*_ = .24, .43). For anxiety, the simple slope was .29 (*SE* = .04; *CI*_*95%*_ = .22, .37; *p* < .0001), with standardized effect size of .37 (*CI*_*95%*_ = .27, .46). For stress, the simple slope was .22 (*SE* = .03; *CI*_*95%*_ = .16, .29; *p*<.0001), with standardized effect size .31 (*CI*_*95%*_ = .21, .40).

**Figure 1.**
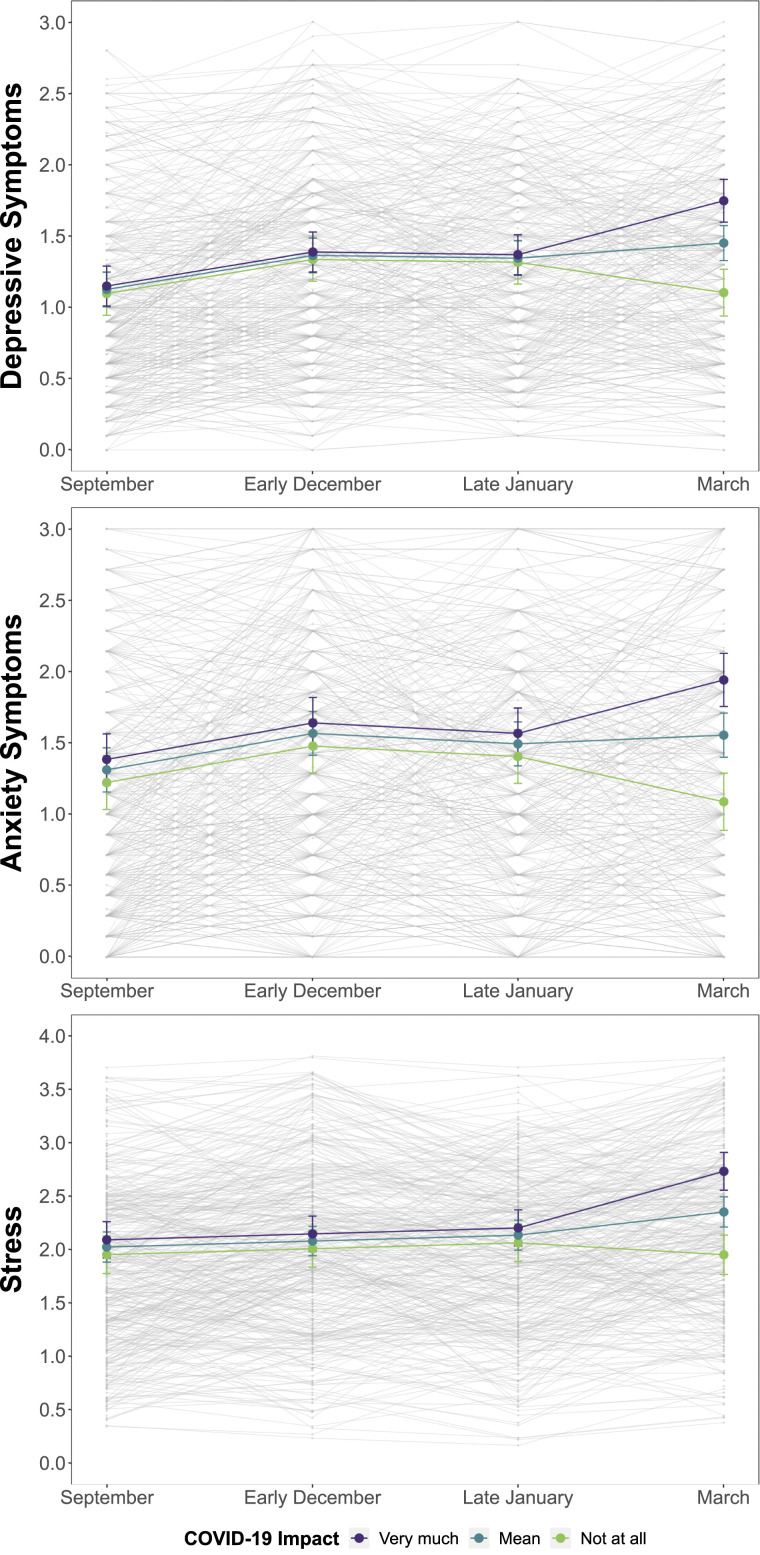
Mean Trajectories of Depression, Anxiety, and Stress from September 2019 to March 2020 and Perceived End-of-Semester COVID-19 Disruption. *Note*. Lines include 95% confidence intervals of symptom estimates at each wave. Observed data for each participant appears in the background.

Standardized effects indicate that a one *SD* increase in reported impact of COVID-19 was associated with an increase in symptoms of .31 *SD*s for stress, .33 *SD*s for depression, and .37 *SD*s for anxiety. The confidence limits show that each of these obtained effects met, but did not exceed, our hypothesized benchmark of .29 and we can also conclude that these effects are statistically different from zero. Figure 1 shows depression, anxiety, and stress trajectories across the year and COVID-19 disruption to those trajectories for students who rated the impact of COVID-19 as “not at all,” at the mean, and “very much.”

### Hypothesis 2a: Similar Mean Trajectories Across Living Arrangements

This hypothesis was supported. Students living with parents had the nominally highest overall mean scores (depression: *M *= 1.25, *SD *= .67; anxiety: *M *= 1.29, *SD *= .85; stress: *M *= 2.04, *SD *= .78) compared to other living arrangements (campus residence: *M *= 1.19, *SD *= .67 for depression, *M *= 1.22, *SD*=.82 for anxiety, and *M *= 1.98, *SD *= .76 for stress; off-campus: *M *= 1.23, *SD *= .56 for depression, *M *= 1.18, *SD *= .77 for anxiety, and *M *= 1.92, *SD *= .68 for stress). Our analysis did not detect significant differences in either mean levels or trajectories.

### Hypothesis 2b: Disruption Worse for Students who Moved

Table 2 shows that 205 participants (40% of the sample) moved residences in March 2020. As expected, the vast majority (94%) of these moves were students leaving residence to live with their parents (*n *= 180) or leaving another independent living situation to live with their parents (*n *= 12). Just two people reported moving *out* of their parents’ homes, and the remaining moves were exchanges between living with to without roommates or vice-versa.

Given the homogeneity of moving reports, and that just 19 people reported moving before March 2020, we retained a single variable coded 0 for students who maintained the same residence and 1 for students who moved in March.

Hypothesis 2b was not supported for any mental health outcome. We added the code for moving and its interaction with each of the three terms that we used to test for the effect of COVID-19 impact in Hypothesis 1b. For depression, the cross-level interaction term was not significant (*Est* = −.02, *SE* = .05; *CI*_*95%*_ = −.11, .08; *p *= .757), indicating that the effect of COVID-19 disruption was not different for people who moved versus maintained the same residence in March 2020. Similar results were found for anxiety (*Est* = .06, *SE* = .06; *CI*_*95%*_ = −.06, .17; *p*=.343) and stress (*Est* = −.06, *SE* = .05; *CI*_*95%*_ = −.17, .04; *p *= .246). In sum, we did not find evidence that COVID-related disruptions in depression, anxiety, or stress symptoms were worse for students who moved in March.

### Hypothesis 2c: Disruption Worse for Financially Vulnerable Students

Students in this sample were socioeconomically diverse. Table 1 shows that mean parent income in this sample was 8.68 (*SD *= 2.48) for students reporting on two parents’ incomes, corresponding to an estimated $117,000 annually, and 5.09 (*SD *= 2.07) for students reporting on one parent’s income, corresponding to an estimated $36,350 annually^2^. The estimate for two-parent families is close to the 2015 provincial median income for couples with children ($110,935) and below the corresponding median income from the same year for single-parent families ($49,428). As reported earlier, 15.7% of students in this sample were food insecure.

With respect to family income, Hypothesis 2c was not supported for any mental health outcome. We included parent income as a mean-centered predictor in the analysis and its interaction with each of the three terms used to test for the effect of COVID-19 impact. For depression, the cross-level interaction term was not significant (*Est* = .001, *SE* = .01; *CI*_*95%*_ = −.02, .02; *p *= .874), indicating that the effect of COVID-19 disruption was not different for people from lower versus higher income families. Similar results were found for anxiety (*Est* = .01, *SE* = .01; *CI*_*95%*_ = −.01, .03; *p *= .296) and stress (*Est* = −.01, *SE* = .01; *CI*_*95%*_ = −.03, .01; *p *= .296). In the absence of tests for interaction, we also found no main effect of parent income, suggesting that after adjusting for other measures including food insecurity, levels of depression, anxiety, and stress were similar on average, regardless of income.

**Figure 2.**
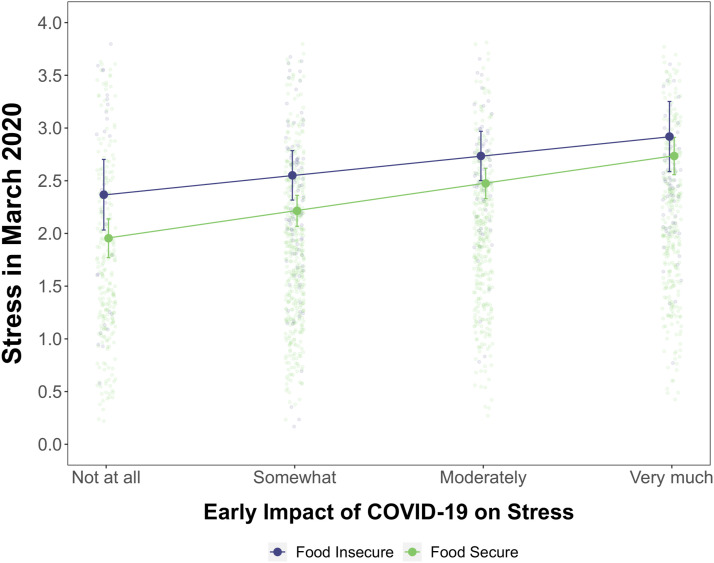
Association between students’ perceptions of the effect of COVID-19 impact on their stress ratings in March 2020 for students classified as food secure and food insecure. *Note*. Lines include 95% confidence intervals of stress estimates at each COVID-19 impact rating. Observed data for each participant appears in the background.

With respect to food insecurity, Hypothesis 2c was not supported for depression or anxiety and inconclusively supported for stress. We included food insecurity as a dummy-coded predictor (0 = food secure and 1 = food insecure) along with its interaction with each of the three terms used to test for the effect of COVID-19 scores. The cross-level interaction terms were not significant for depression (*Est* = −.13, *SE* = .08; *CI*_*95%*_ = −.29, .02; *p *= .087) or anxiety (*Est* = −.04, *SE* = .09; *CI*_*95%*_ = −.23, .14; *p*=.650), but were significant for stress (*Est* = −.19, *SE* = .09; *CI*_*95%*_ = −.35, −.03; *p *= .019). However, when the model excluded the corresponding interaction for parent income, the COVID-19 impact × food insecurity interaction was no longer significant after correction for multiple testing (*Est* = −.16, *SE* = .08; *CI*_*95%*_ = −.31, −.01; *p *= .034 against a Benjamini–Hochberg FDR-adjusted threshold of *p*<.029). Figure 2 shows the early impact of COVID-19 on stress in March 2020 for students classified as food secure and food insecure. The source of the inconclusive interaction is also opposite to our hypothesized effect: the effect of perceived COVID-19 impact was marginally stronger for *food secure* students. Students experiencing food insecurity reported stress levels that were already higher even if they reported no perceived COVID-19 impact and increased more gradually with increasing impact of COVID-19. However, we reiterate that this effect did not meet our null hypothesis inference criteria and the confidence intervals overlap in Figure 2.

For all mental health outcomes, we found a robust main effect indicating that on average, students experiencing food insecurity at the start of their transition to university were more depressed, more anxious, and more stressed than their food secure counterparts. Partially standardized effects indicate that the difference between food secure and insecure groups was .48 *SD*s for depression (*CI*_*95%*_ = .27, .70) and .30 *SD*s for anxiety (*CI*_*95%*_ = .08, .52). When the COVID-19 × food insecurity interaction is excluded from the model predicting stress, the difference between food secure and insecure groups was .43 *SD*s (*CI*_*95%*_ = .22, .65). In sum, students reporting food insecurity during the transition to university also reported poorer mental health, compared to food secure students, but perceived COVID-19 impact on mental health was not worse for students reporting food insecurity.

### Exploratory Aim 1: Interactions Between COVID-19 Disruption, Living Situation, and Financial Vulnerability

We added interactions between parent income and living away from home, parent income and living in residence, food insecurity and living away from home, and food insecurity and living in residence as well as interactions between each of these terms with the three terms used to test for the effect of COVID-19 impact on each mental health outcome. None of the highest-order interaction terms were statistically significant in any model, indicating that effects of living situation and financial vulnerability were additive, not multiplicative, in their associations with COVID-related disruptions to end-of-semester depression, anxiety, and stress.

## Discussion

This study modeled trajectories of depression, anxiety, and stress across the first year of university and found robust evidence that stronger ratings of the subjective impact of the COVID-19 pandemic were associated with unusually elevated symptoms at the end of the academic year. We hypothesized that disruptions to mental health would be amplified for students who moved residences in March and for students who were more financially vulnerable. Results did not support these hypotheses. Below we discuss key findings emerging from this study.

### Mental health trajectories were typical across students’ first year until the onset of the pandemic

Consistent with Hypothesis 1a, trajectories of depression and anxiety in Figure 1 show the characteristic rise in symptoms through students’ first semester followed by stability seen in other longitudinal samples (e.g., Barker et al., 2018; Conley et al., 2014; Cooke et al., 2006). The mean trajectory of stress increased linearly rather than leveling off after students’ first semester. As expected, the transition to university in the Fall of 2019 was associated with increases in mental health difficulties. Most students also reported that their responses to depression, anxiety, and stress questionnaires administered in March 2020 were at least slightly influenced by feelings and reactions they were having to the incipient COVID-19 pandemic. Consistent with Hypothesis 1b, stronger feelings of COVID-19 impact corresponded with more mental health symptoms. Figure 1 showed that symptoms remained stable or worsened for students who reported COVID-19 impact that was at the mean or higher for each measure, and Table 1 shows that mean scores on all measures were highest in March. In contrast, in a typical academic year, symptoms tend to remain stable or improve toward the end of the year (e.g., Barker et al., 2018; Hirai et al., 2015). Whether these unfavorable trajectories translate to poorer long-term outcomes such as higher rates of university dropout and more diagnosable depression and anxiety remains to be seen.

The level of disruption associated with students’ perceptions of COVID-19 impact in the present study is comparable to disruption observed in other settings in which young people navigate a meaningful developmental transition. Indeed, we found that the magnitudes of perceived COVID-19 impact on students’ depression, anxiety, and stress were as large as the magnitude of change in mental health observed in students assessed before and after they begin their first year of university (Conley et al., 2014; Cooke et al., 2006; Doane et al., 2015; Levine et al., 2020). Crucially, COVID-related disruption to students’ mental health in the present sample captures the very earliest signs of pandemic impact on mental health. Students in the present sample were assessed within the first weeks of pandemic-related disruption in Canada. Locally, activities had been business-as-usual up until one week before the March survey became available to participants. This is in stark contrast to a study of undergraduates in New York City who were surveyed around the same time but after living at least six weeks under shelter-at-home orders (López-Castro et al., 2021), of whom 17% reported the death of a family member or close friend to COVID-19. Clinically concerning symptoms of depression and anxiety were evident in 90% and 66% of students, respectively. A longitudinal study following undergraduates in Guangdong Province, China, similarly found increases in depression and anxiety two months after widespread pandemic closures (Li et al., 2021). In the present sample, student mental health may have continued to worsen in the weeks after our survey was complete.

### The early impact of COVID-19 was not worse for students who moved residences

Consistent with Hypothesis 2a, students living at home, in residence, and off-campus had similar mean levels and trajectories of depression, anxiety, and stress. We found that 40% of students moved residences in March, most of whom left campus residence to live with their parents. Contrary to Hypothesis 2b, however, we found that the effects of students’ feelings of COVID-19 impact on their depression, anxiety, and stress were similar for students who moved and who didn’t.

This finding contradicts past studies showing that emerging adults who move back into their parents’ homes report worse mental health (Caputo, 2020; Copp et al., 2017) but is consistent with recent work surveying U.S. undergraduates forced to relocate out of campus residence in the spring of 2020 (Conrad et al., 2021). In that study, students also largely moved back into parents’ homes and moving was unrelated to depression or post-traumatic stress, and only weakly associated with higher anxiety. Another study found that poorer mental health in students who moved back into parents’ homes following COVID-related campus closures was linked to factors like feeling a lack of autonomy at home, negative interactions with parents, and not wanting to live at home (Hall & Zygmunt, 2021).

Campus residence is a uniquely semi-dependent living situation featuring less responsibility, easier access to resources (Beiter et al., 2015), and for many, continued financial dependence on parents (López Turley & Wodtke, 2010) compared to fully independent living. Living situations classified as independent in previous studies were more diverse and assessed in samples of primarily older emerging adults. Students in the present sample who moved in March were all under 20 years old and had been living in campus residence for less than 7 months. Had there been no COVID-related disruption, students would have begun moving out of campus residence within a month at the conclusion of the academic year—and for most, back into their parents’ homes. Pandemic conditions notwithstanding, residential mobility was likely experienced more as an accelerated timeline than a mass disruption for many students in this sample.

Another consideration is that the shared context of pandemic disruption meant all students were experiencing similar setbacks, uncertainties, and difficult decisions. In other words, disruption was normative, and students leaving residence were all moving over the same compressed period of time. In previous studies, residential mobility occurred at different times and for individual reasons (Caputo, 2020; Copp et al., 2017). Given the shared context of the pandemic, there may have been comfort in the knowledge that peers were enduring similar challenges. In some respects, residential mobility brought on by COVID-19 represented a common contextual transition rather than an individual deviation from social norms and expectations. Feelings of psychological distress that accompany perceptions of unfulfilled societal expectations (Culatta & Clay-Warner, 2021) may have thus been diminished or absent. And indeed, feelings of solidarity and community may have been heightened (Di Napoli et al., 2021).

### The early impact of COVID-19 was not worse for financially vulnerable students

Overall, our models did not support Hypothesis 2c that the early impact of COVID-19 was worse for financially vulnerable students. Students reporting food insecurity had persistently higher levels of depression, anxiety, and stress during their first year of university compared to students who were food secure, by as much as .48 of a *SD*. We also verified that students reporting lower parent incomes had higher levels of depression, anxiety, and stress but these effects were only evident in models that excluded food insecurity (effect sizes for parent income were a third the size of effects for food insecurity, or less). Despite persistent mental health disadvantages for financially vulnerable students, their response to the onset of the COVID-19 pandemic was similar to their better-resourced peers.

Several conditions of the present study may account for why students experiencing greater financial vulnerability did not, as a group, report proportionally worse mental health at the onset of the pandemic. First, at the launch date of the March survey, the Province of Ontario had recorded just 377 COVID-19 cases and 2 deaths in a population exceeding 14 million (Government of Ontario, 2020c). At this point, most if not all students in the present sample would have experienced major changes to daily life but no direct or personal impact (e.g., COVID-related illness or death in the family).

Second, on March 25 the federal government formally announced the *Canada Emergency Response Benefit* (CERB) taxable stipend of $2000 per month to anyone not receiving ordinary employment insurance benefits after job loss that would be retroactive to March 15 (Department of Finance Canada, 2020). Anticipation of a universal basic income may have mitigated some of the incremental distress we might otherwise have expected to observe in students from more financially vulnerable households. Data gathered in the summer of 2020 from a sample of 22-year-olds in the province of Québec showed that most emerging adults were not very concerned about being able to meet their basic needs, and mental health scores were not significantly elevated relative to an assessment two years prior (Watkins-Martin et al., 2021). Whether social safety net policies protect mental health as well as economic security during public emergencies is a question worthy of further study.

Finally, students in this sample were all under age 20, meaning that relatively few were likely serving as heads-of-household responsible for all financial obligations. Indeed, 81% of the 408 students who provided data in March were living with parents.

#### Study Limitations

Our findings are limited to the perceived impact of COVID-19 on student mental health at the start of the pandemic, before COVID-19 became severe in Canada. The mental health levels found in the March 2020 wave can only be attributed to early situational features of COVID-19 (e.g., online learning, unemployment, and residential relocation) and not to the challenges endured as COVID-19 progressed (e.g., death of a loved one, testing positive or becoming ill, prolonged social isolation, and sheltering in a neighborhood with higher case counts). The present study also lacks generalizability as our first-year undergraduate sample was drawn from a single Canadian university.

## Conclusion

The results of the current study show that mental health outcomes during the early part of the pandemic were greatest for those who reported the largest subjective COVID-19 impact. These students showed increases in mental health symptoms at a point in the academic year when symptoms typically plateau or decline, and this effect was comparable in size to mental health disruptions typically seen among students entering university. How students responded to the initial disruptions associated with the pandemic did not depend on financial capacity and did not differ for students who chose to relocate. These null findings suggest that emotional and tangible supports afforded by moving in with parents and financial support from government programs may have offset further increases in distress. Findings highlight the importance of wider contextual supports for university students’ well-being and adjustment.

## Notes

1 Data for this study had already been collected but were not examined prior to acceptance of our Stage 1 report, with the exception of data from September 2019, used in another project to assess differences in mental health for students with and without food insecurity (Howard & Barker, 2021)

2 The procedure used to compute estimated incomes for descriptive purposes based on incomes reported from ordinal categories is documented in Howard et al., 2020.
